# Treatment with tofacitinib attenuates muscle loss through myogenin activation in the collagen-induced arthritis

**DOI:** 10.1186/s42358-024-00416-5

**Published:** 2024-11-13

**Authors:** Thales Hein da Rosa, Bárbara Jonson Bartikoski, Rafaela Cavalheiro do Espírito Santo, Mirian Farinon, Jordana Miranda de Souza Silva, Renata Ternus Pedó, Maria Luísa Gasparini, Thais Karnopp, Leonardo Peterson dos Santos, Gustavo Chapacais, Andressa di Domenico, Sofia Loch, Ricardo Machado Xavier

**Affiliations:** 1 Laboratório de Doenças Autoimunes, Serviço de Reumatologia, Hospital de Clínicas de Porto Alegre, Rua Ramiro Barcelos, 2350, Porto Alegre, RS 90035-903 Brazil; 2 Programa de Pós-Graduação em Ciências Médicas, Faculdade de Medicina, Universidade Federal do Rio Grande do Sul, Ramiro Barcelos, 2400, Porto Alegre, RS 90035-003 Brazil; 3 Health Research and Innovation Science Centre, Klaipeda University, Klaipeda, Lithuania

**Keywords:** Muscle loss, Rheumatoid arthritis, Janus Kinase inhibitor, Collagen-induced arthritis, Myogenin

## Abstract

**Background:**

Sarcopenia is a muscle disease characterized by reduction of muscle strength and muscle mass. In RA, 25.9 to 43.3% of the patients present sarcopenia. The loss of muscle mass observed in RA patients occurs either by activation of catabolic pathways or by inhibition of anabolic pathways. Despite having a list of drugs capable of treating RA inflammation, their effect on muscle is unclear. Our objective was to evaluate the tofacitinib effect on the muscle mass of collagen-induced arthritis (CIA) mice.

**Methods:**

CIA was induced in male DBA/1J mice by subcutaneous injection of Type 2 Collagen plus Freund Adjuvant. Animals were randomized into 3 groups: CIA + tofacitinib; CIA + vehicle; and healthy controls. Treatment was administered twice a day, between days 18 and 45 after induction. Clinical score, edema, and body weight were evaluated during the experimental period. After euthanasia, tibiotarsal joints were collected for assessment of disease histopathological score, and tibialis anterior (TA) and gastrocnemius (GA) muscles were weighed to assess muscle mass. Muscle atrophy was evaluated by measurement of TA myofiber cross-sectional area (CSA). Protein expression was evaluated by western blot using GA homogenates. Serum inflammatory markers were evaluated by ELISA. Statistical analysis included ANOVA followed by Tukey’s or with Kruskal-Wallis. The statistical difference was assumed for *p* < 0.05.

**Results:**

Tofacitinib treatment decreased arthritis severity by reducing clinical score, and hind paw edema in comparison with the vehicle group. Tofacitinib showed weight gain, higher TA and GA weights, and increased CSA compared to the vehicle group. On day 45, Tofacitinib presented increased muscle strength compared to the vehicle group, however, no difference was found in muscle fatigue. Pax7 expression was unchanged, while MyoD expression showed an increasing trend, and myogenin expression was significantly increased in Tofacitinib compared to vehicle and control groups. The treatment didn’t modify Murf-1 expression. Tofacitinib mice showed decreased serum levels of TNF and increased IL-6 serum levels.

**Conclusion:**

Tofacitinib attenuated muscle loss in arthritic mice, increased muscle weight and muscle CSA. Activation of satellite cell regeneration, based on the increased expression of myogenin, is a potential mechanism involved in tofacitinib action against muscle loss.

**Supplementary information:**

The online version contains supplementary material available at 10.1186/s42358-024-00416-5.

## Introduction

Rheumatoid arthritis (RA) is a chronic, autoimmune, systemic disease that leads to joint destruction. RA systemic inflammation may also result in comorbidities such as metabolic syndrome [1], cardiac disease [2], and sarcopenia [3]. Sarcopenia is a muscle disease characterized by loss of muscle strength followed by loss of muscle mass, which may impact physical function [3]. It is known that loss of muscle mass in RA patients is driven by the activation of catabolic pathways and/or by the inhibition of anabolic pathways [4]. In the same way, muscle strength and contractility reduction in RA occurs possibly by an inflammatory elevation of reactive oxygen and nitrogen species (ROS/RNS) [5] and is related to a consequent impairment of quality of life [6].

Pro-inflammatory cytokines, such as interleukin-1β (IL-1β), interleukin-6 (IL-6), and tumor necrosis factor-α (TNF-α), are highly produced in RA and can promote an increase in autophagy rate and activate ubiquitin proteasomal system (UPS) in muscle cells [4, 7]. Additionally, inflammatory mediators decrease the activation and differentiation of satellite cells, responsible for myogenesis, also leading to muscle atrophy [4]. During quiescence, satellite cells express the Paired Box Transcription Factor 7 (Pax7) [8, 9] and, to promote muscle regeneration, they undergo a process controlled by the sequential expression of transcription factors [10]. Therefore, satellite cells have great myogenic potential, being dependent on Pax7 expression and later on myogenic regulatory factors (MRFs), such as the muscle-specific proteins Myogenic Differentiation 1 (MyoD), Myogenic Factor 5 (Myf5), Myogenin, and Myogenic Regulatory Factor 4 (MRF4) [11]. Myogenin, together with MyoD and MRF4, activates the myogenic differentiation program.

Tofacitinib is a Janus Kinase inhibitor (JAKi), which inhibits the activity of JAK1 and JAK3 and, to a lesser extent, JAK2 and TYK2 [12]. The binding of cytokines such as IL-2, IL-4, IL-6, IL-7, and type I and type II interferon (IFN) to their receptors, on the cell surface membrane, activates JAKs to phosphorylate the signal transducer and activator of transcription (STATs), that translocates to the nucleus to regulate gene expression [13, 14]. In muscle cells, the STATs pathway is able to activate protein degradation and myogenic inhibition [15–17]. Indeed, the inhibition of JAK/STAT pathway was related to muscle function restoration in a mice model of critical illness myopathy, a model that subjects animals to impaired muscle weight and function [18].

Despite having a list of drugs capable of reducing disease activity, there are no approved therapeutic interventions that prevent or slow the development of sarcopenia in RA patients [19]. Additionally, a treatment that is able to stimulate the muscle regeneration process is sought. As it remains unclear whether JAKi therapy is able to influence the muscle loss or regeneration process in RA, our objective was to evaluate the effect of tofacitinib on the muscle mass of collagen-induced arthritis (CIA) mice.

## Methods

### Animals

Male DBA/1J mice were used. During experimentation, animals were maintained in a 12 h dark/light cycle, 20 to 24 °C temperature, and 40–60% humidity level. Water and food were administered *ad libitum.* All experiments were performed following the Guiding Principles for Research Involving Animals. This study was approved by the Research Ethics Committee of the Hospital de Clínicas de Porto Alegre (protocol number 18-0302). The study complies with the principles of the 3Rs: Replacement of animals by alternatives wherever possible, Reduction in the number of animals used, and Refinement of experimental conditions and procedures to minimize the harm to animals. All animals were anesthetized with isoflurane (Abbott, Abbott Park, IL, USA) during arthritis induction and euthanasia and were monitored twice a week to assess any behavioral change and loss of quality of life, observing signals of inactivity, suffering, stress, unbearable pain and no food or water intake.

### Collagen-induced arthritis model

**Fig. 1:**
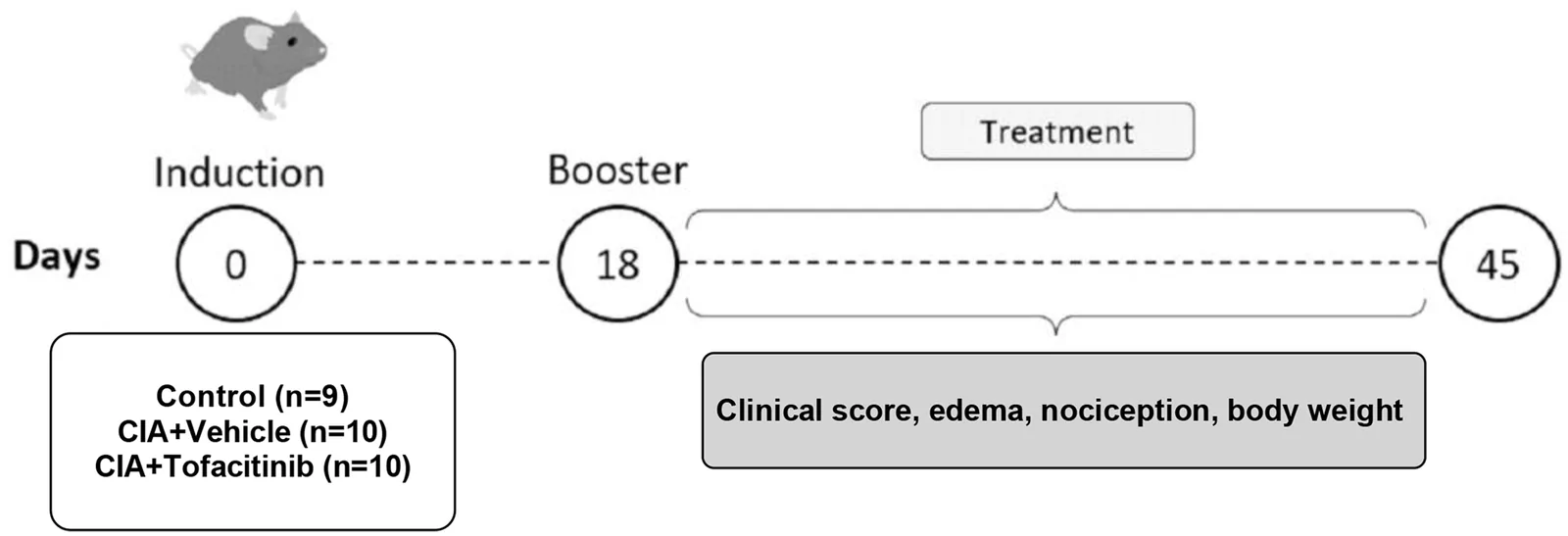
Experimental method during 45 days of disease induction

CIA was induced by 2 mg/ml of bovine type II collagen plus complete Freund’s adjuvant intradermal injection at the tail at day 0. At day 18, mice received a booster injection with bovine type II bovine collagen and incomplete Freund’s adjuvant. Animals were randomized into three groups: healthy mice without intervention (control group), arthritis-induced animals treated subcutaneously with PBS (vehicle group), and arthritis-induced animals treated with tofacitinib (tofacitinib group) at the dosage of 30/mg/kg/day (diluted in PBS 1X). The treatment with tofacitinib was subcutaneously administered, twice a day, from day 19 after CIA induction to day 45 (27 days of treatment) (Fig. 1). The dosing strategy was planned according to the literature [12] and the administration was decided in order to respect the short half-life of tofacitinib. Forty-five days after disease induction, animals were anesthetized with isoflurane and euthanized by cervical dislocation. Ankle joints were collected for confirmation of arthritis induction by histological analysis. In addition, serum samples were collected, and the muscles tibialis anterior (TA) and gastrocnemius (GA) were dissected, weighted, and processed for histological and molecular analyses, respectively.

### Clinical score

Animals were monitored daily during the whole experimental period by blinded evaluators, for clinical signs of arthritis according to a severity score: 0) without erythema and edema; 1) light edema and erythema; 2) mild edema and erythema; 3) severe edema and erythema in metatarsal and ankle joints; 4) all joints affected by edema and erythema and loss of function (ankylosis). Total animal severity scores were considered by the sum of each paw score, with being 16 the highest score [20]. The arthritis clinical score was measured by a blinded examiner.

### Paw edema

Paw edema was measured in animals’ hind paws. This measure was performed by the submersion of animal paws in equipment called plethysmometer (Insight Ltda., Ribeirão Preto, Brazil). This equipment measures the dislocation of volume attributed to edema in animal paws [20]. Edema was measured by a blinded examiner.

### Physical performance

A physical performance test was held to analyze the time of fatigue at days 0, 18, 25, 35, and 45 after CIA induction. Physical performance tests were done in the morning, at the same time period, and by the same evaluators. For the physical performance test, animals were initially placed on the treadmill for 5 min of ambiance before the test started. The treadmill started at 8.5 m/min speed and was raised by 2.5 m/min every 3 min reaching the maximum of 45 m/min. Animals were removed from the treadmill when exhausted, defined by the incapacity to keep running. Performance was estimated using two parameters: total duration of running (time in minutes) and distance traveled (meters). The distance was used as the main parameter to estimate resistance performance. Fatigue assay was performed by a blinded examiner.

### Muscle strength

The maximum grip strength test was adapted from Deacon et al. [21]. Meshes with proper loads, each one amounting to 5, 20, 35, 50, 65, 80, and 95 grams were used. The mouse was held by the tail and suspended until it grasped the lighter weight with all paws for at least 3 recorded seconds. If the animal succeeded, it rested for the 30s before trying the next weight. If the animal failed three times with a 10s rest between each attempt, the longest time it was able to hold the weight was recorded. The following equation was used: Fmax = P3seg + (5*t < 3seg), where Fmax is the maximum calculated grip strength, “P3seg” is the heaviest load the animal held for 3 s, and “t < 3seg” is the longest time the animal held the heaviest load. The final result was expressed in grams (g) [22]. Grip strength assay was performed by a blinded examiner.

### Nociception

Hind paw nociception was evaluated at days 0, 18, 25, 35, and 45 after CIA induction. Mice were placed individually in acrylic boxes. The experiment was performed by a blinded examiner, using an analgesimeter that presses the hind paw of mice and registers the intensity of the force applied when the animal withdraws its paw [23].

### Joint histopathology

After anatomic dissection, joints were fixed in 10% formol for 72 h, decalcified in 10% nitric acid for 24 h, paraffin was embedded, sectioned, and stained with hematoxylin-eosin (HE). HE slides were analyzed by a blinded pathologist to establish the histopathological score [24].

### Muscle weight and myofiber cross-sectional area

The TA and GA muscle weights were obtained after euthanasia and organ dissection. TA muscle was immersed in 10% buffered formalin for fixation for up to 2 days. The samples were dehydrated and paraffin-embedded. Slices 6 μm thick were arranged on microscope slides and HE stained using a standard protocol (Fischer et al. [25]). Myofiber cross-sectional area (CSA) measurement was determined using 10 pictures of each muscle sample, and 20 fibers of each picture were sized using the Image-Pro Express software (version 5.1.0.12, Media Cybernetics, Rockville, MD, USA) and counted by two blinded examiners.

### Western blot analyses

Western blot analyses were performed as previously described [26]. Briefly, TA muscles were homogenized in a lysis buffer containing protease inhibitors (cat# A32955, Thermo Scientific) and phosphorylation protease inhibitors (cat# 4906845001, Roche). After quantification of protein concentrations using the Bradford method, the lysates or total proteins (25 μg/lane) were separated by SDS-PAGE on 12–16% gels and transferred onto polyvinylidene fluoride (PVDF) membrane (cat# IPFL00010, Millipore, Bedford, Mass). After being blocked with 5% milk, the membranes were probed with primary antibodies overnight at 4 °C. The primary antibodies used in this study were myogenin (1:1000, Sigma-Aldrich (SAB2501587, lot 9955P1)), MyoD (1:1000, Sigma-Aldrich (SAB4300397, lot 8715), Pax7 and GAPDH (1:1000, Sigma-Aldrich (G9545, lot 092M4800V)). Finally, the detection was made by the binding of the secondary antibody with the primary antibody, leading to a chemiluminescent signal. All raw data is available in the supplementary material file (Fig. 3).

### Enzyme-linked immunosorbent assay

Serum samples were used for TNF-α (ELISA MAX Deluxe Set (430904) BioLegend, San Diego, CA, United States) and IL-6 (Mouse ELISA MAX Deluxe Set (431304), BioLegend, San Diego, CA, United States) detection by enzyme-linked immunosorbent assay (ELISA) according to the manufacturer’s instructions.

### Statistical analysis

The sample size was calculated based on the previous study of our group, in which muscle atrophy was assessed in CIA mice by the myofiber CSA measurement area [27]. The authors found a 26 and 31% (*p* < 0.05) decrease of GA and TA myofiber CSA, respectively, in CIA group compared to CO group 45 days after disease induction. Therefore, considering this difference and using a power of 80% and a confidence of 95%, the sample size resulted in n of 9 animals per group. Considering the possibility of animal death due to error in the intradermal injection or the need for anticipation of death, 1 animal was added per group (rate 10%). So, our sample size was 10 mice per group. After confirmation of Gaussian distribution by Shapiro–Wilk and Kolmogorov–Smirnov tests, quantitative data were described as mean ± standard error of the mean (SEM). Comparison between groups was performed using one-way Analysis of variance (ANOVA) followed by Bonferroni’s test or Tukey’s test and two-way ANOVA followed by Bonferroni’s test or Tukey’s. All statistical tests were performed in GraphPad Prism software. Differences were considered statistically significant when *p* < 0.05.

## Results

### Tofacitinib treatment decreased clinical score and edema in collagen-induced arthritis mice

**Fig. 2:**
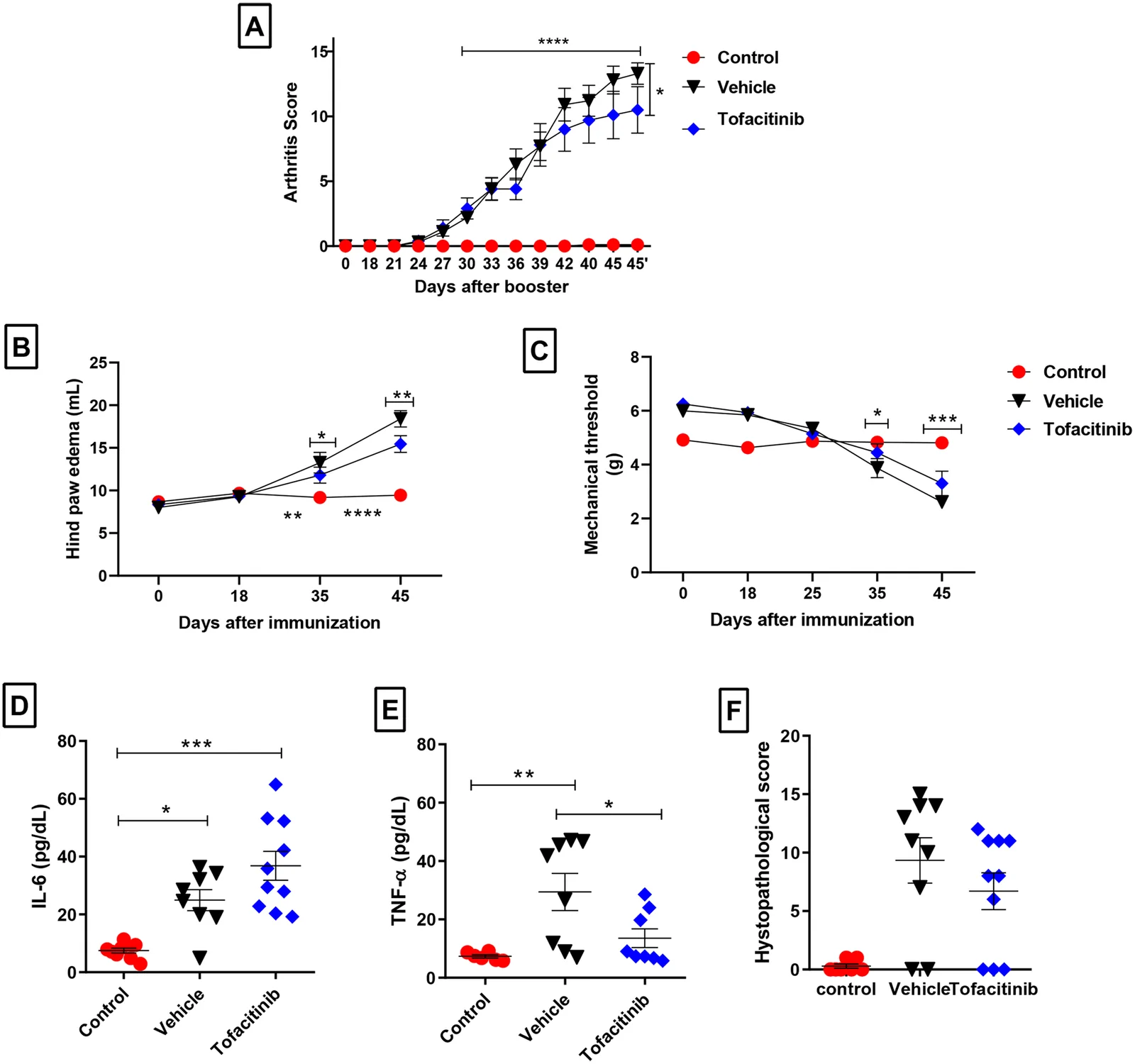
CIA mice developed experimental arthritis and high disease severity. **A** Arthritis score and **B** Hind paw edema decrease over time in the tofacitinib-treated group compared to the vehicle group. **C** The mechanical threshold of nociception was decreased in both vehicle and tofacitinib groups compared to control mice. **D** Serum protein expression of IL-6 and **E** TNF-α from all experimental groups. **F** Histopathological score. Arrows indicate immune cell infiltration and bone/cartilage erosion. Representative data over time is related to established CIA at 50 days of disease. Data are presented as mean ± standard error of the mean (SEM). Statistical analysis between groups was performed using a two-way analysis of variance followed by Bonferroni’s test (Arthritis score, hind paw edema, nociception) and ANOVA one-way followed by Bonferroni’s test (Histopathological score, IL-.6, TNF-α). *p < 0.05; **p < 0.01; ***p < 0.0001. Control group n = 9; Vehicle group n = 10; Tofacitinib group n = 10

CIA model was performed for 45 days in total, being the induction marked as day 0 and the beginning of the treatment on day 18. During animal experimentation, 3 mice died before the euthanasia day. Mice subjected to CIA successfully developed the disease as they presented increased clinical arthritis score (*p* < 0.001; Fig. 2A), and hind paw edema (*p* < 0.001; Fig. 2B) compared to the control group. The histopathology of joints also reveals the presence of synovial inflammation, hyperplasia, *pannus* formation, cartilage, and bone erosion in CIA mice compared to the control group (Fig. 2F, Supplementary Fig. 1).

**Fig. 3:**
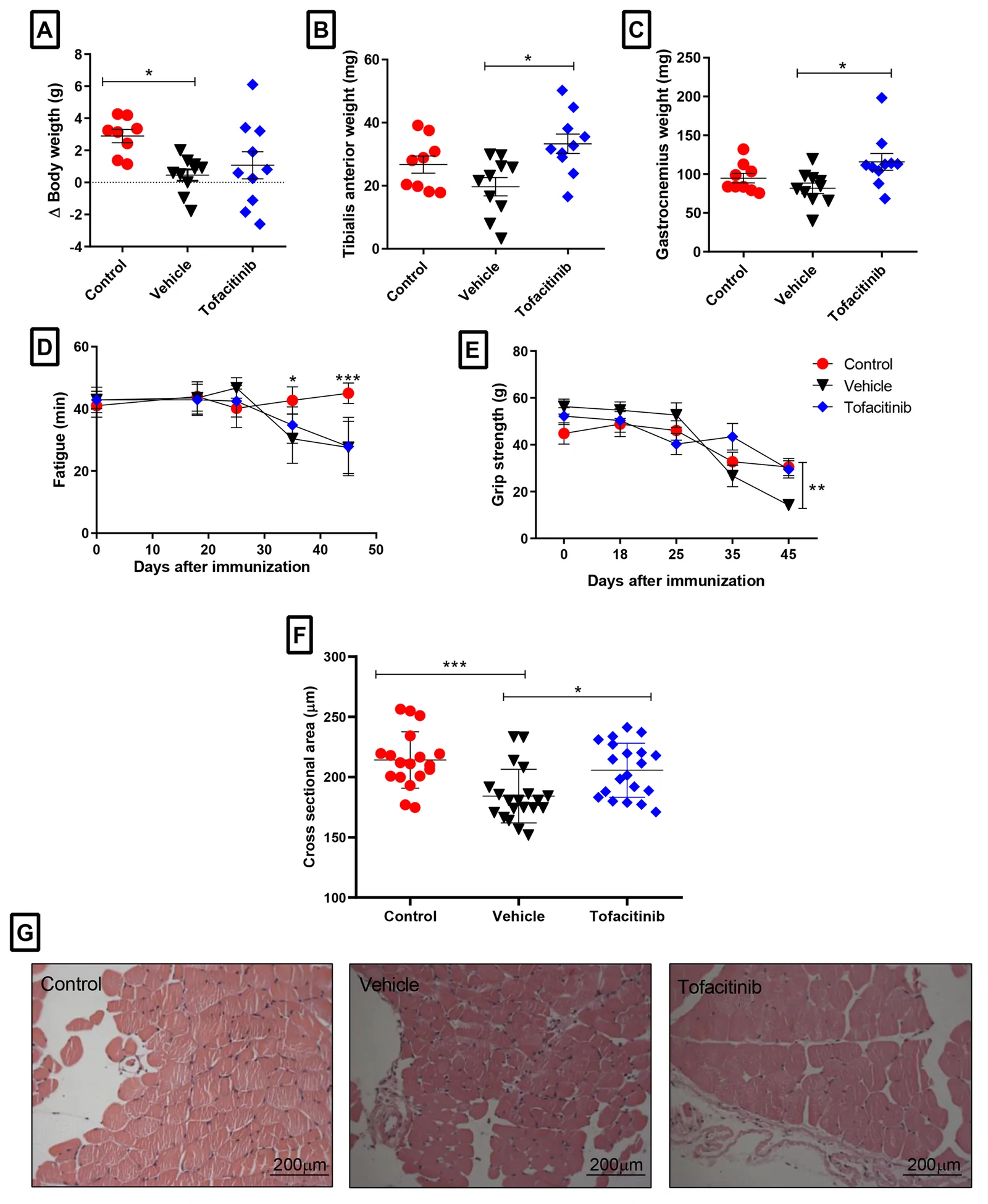
Tofacitinib treatment increases muscle quality by present higher muscle weight, grip strength, and myofiber cross-sectional area compared to the Vehicle group. **A** Cumulative delta body weight showed no change during tofacitinib treatment compared to the control group. **B** TA muscle weight and **C** GA weight was higher in the tofacitinib group when compared with the vehicle group. **D** Fatigue represented by the endurance performance test showed an increase in both the tofacitinib and vehicle group compared to the control group. **E** Grip strength and **F** CSA of TA muscle presented increased values in the tofacitinib group compared to the vehicle group. **G** Representative images of muscle sections. Data are presented as mean ± standard error of the mean (SEM). Statistical analysis between groups was performed using ANOVA one-way followed by Bonferroni’s test (Delta body weight, TA weight, GA weight, myofiber CSA) and ANOVA two-way followed by Bonferroni’s test (fatigue, grip strength). *p < 0.05; **p < 0.01; ***p < 0.001. Control group n = 9; Vehicle group n = 10; Tofacitinib group n = 10

On day 45 tofacitinib-treated mice showed a significantly decreased clinical score (*p* = 0.03; Fig. 2A) and decreased paw swelling (*p* = 0.04; Fig. 2B), compared to the vehicle-treated mice on day 45. Interestingly, despite improved clinical and histopathological joint scores, tofacitinib treatment did not change nociception in CIA mice compared to control and vehicle-treated groups (Fig. 2C).

### Tofacitinib decreased serum levels of TNF-α, and increased levels of IL-6 in CIA mice

The levels of TNF-α were decreased in the serum of mice treated with tofacitinib compared to the vehicle-treated group (Fig. 2E) at day 45. On the other hand, IL-6 levels were increased in the tofacitinib group compared to the control group (*p* = 0.0002; Fig. 2D).

### Tofacitinib treatment improved muscle weight, muscle CSA, and muscle strength in CIA mice

Disease animals treated with vehicle had less weight gain compared to the control group (*p* = 0.02; Fig. 3A). We also shown that tofacitinb group had a trend to gain more weight than vehicle treated mice. Regarding muscle mass, the tofacitinib-treated group showed higher TA and GA weights at day 45 compared to the vehicle-treated mice (*p* = 0.009, *p* = 0.02; Fig. 3B and C, respectively).

The induction of arthritis led to muscle atrophy, as a vehicle-treated group showed a lower CSA (decrease of 14%) compared to the control mice (*p* = 0.0004; Fig. 3F). Tofacitinib treatment was effective in decrese muscle atrophy as treated mice showed greater muscle CSA at day 45 (an increase of 11.6%) compared to the vehicle-treated mice (*p* = 0.01; Fig. 3F).

Regarding muscle strength, tofacitinib was able to increase the strength of treated mice on day 45 compared to the vehicle-treated group (*p* = 0.006; Fig. 3E). However, no difference was found in the fatigue time (*p* > 0.05; Fig. 3D). Higher muscle strength, TA, and GA weight were found when these data were normalized by total body weight (Supplementary Fig. 2A, B, and C).

### Tofacitinib promotes muscle cell differentiation and hypertrophy by increasing myogenin expression

**Fig. 4:**
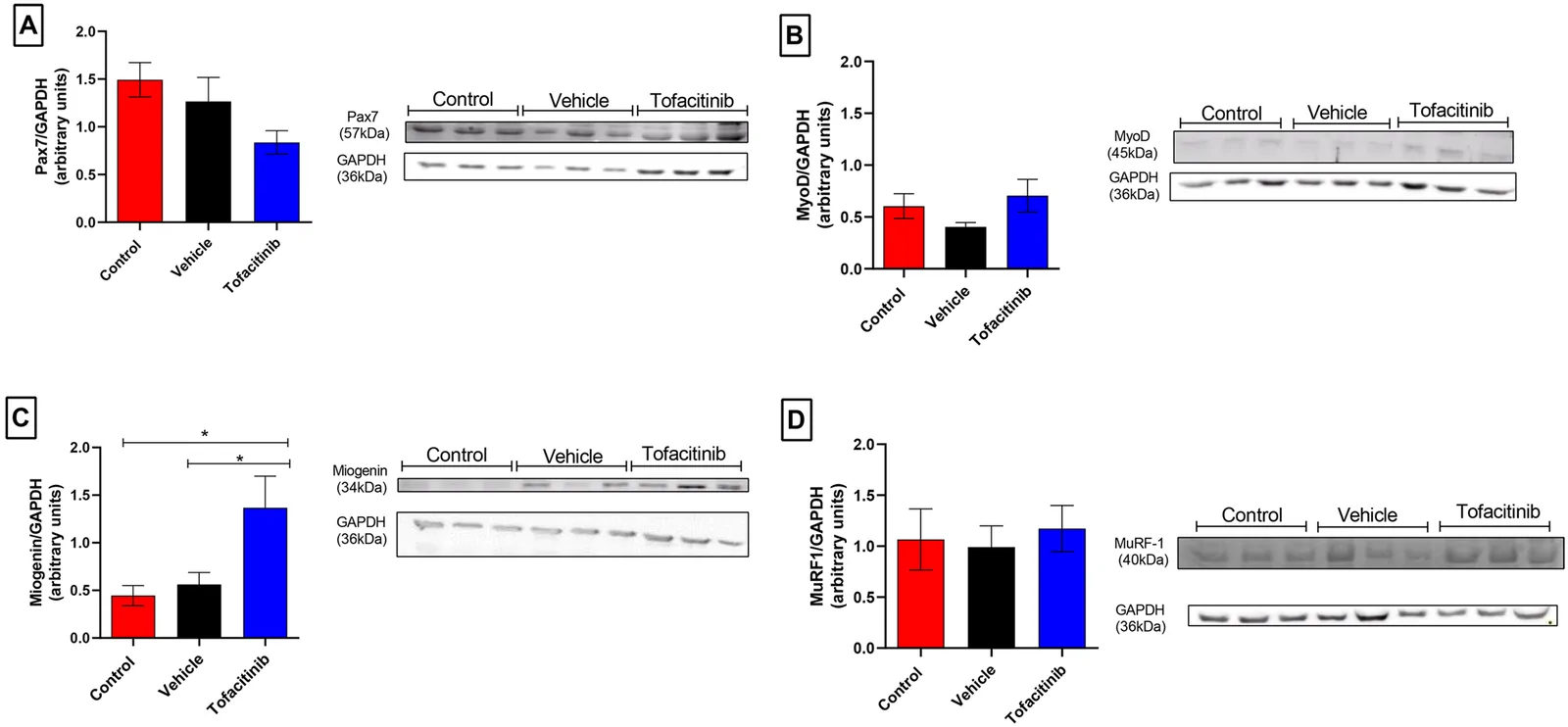
Tofacitinib treatment increases protein levels of myogenin in TA muscle without changes in Myod and Pax7 content. Protein expression of regeneration markers in TA muscle **A** PAX7, **B** Myod, **C** Myogenin, and **D** MuRF-1 with the representative western blot bands. Statistical analysis between groups was performed using ANOVA one-way followed by Tukey’s test. Data are represented as mean ± standard error of the mean (SEM). *p < 0.05. Control group n = 3; Vehicle group n = 3; Tofacitinib group n = 3

We analyzed the protein expression of muscle cell differentiation factors Pax7, MyoD, myogenin, and the catabolic marker MurF-1 (Fig. 4, Supplementary Fig. 3). Tofacitinib treatment does not significantly change Pax7 or MyoD, although there is a trend of lower Pax7 (*p* = 0.07; Fig. 4A), and higher MyoD in the tofacitinib-treated group (*p* = 0.07; Fig. 4B) compared to the PBS-treated group. On the other hand, tofacitinib treatment significantly increased myogenin expression, compared to the vehicle-treated and control groups (*p* = 0.04, *p* = 0.02, respectively, Fig. 4C). Also, the treatment did not significantly modify Murf-1 expression (Fig. 4D).

## Discussion

Muscle wasting is often related to inflammatory diseases and that has been a challenge to physicians. In RA, 25.9 to 43.3% of the patients present sarcopenia, a muscular disease characterized by a reduction of muscle strength and muscle mass. There are several available treatments for RA that aim to attenuate disease activity by blocking inflammatory mediators and their signaling or inducing anti-inflammatory and regulatory pathways [19, 28, 29]. However, their effect on skeletal muscle tissue in RA patients remains unclear. Our objective was to evaluate the effect of tofacitinib on the muscle mass of collagen-induced arthritis (CIA) mice. We demonstrate that treatment with tofacitinib, a targeted synthetic disease-modifying antirheumatic drug (tsDMARDs), was able to modulate the muscle regeneration process in arthritic mice as indicated by increased expression of myogenin. Also, reduced sarcopenia was observed in arthritic mice following tofacitinib treatment, with higher TA and GA weights and concomitant strength improvement.

It is known that the JAK/STATs pathway have an important role in the inflammatory status of RA patients, as they are attached to pro-inflammatory cytokines membrane receptors, and signals downstream transcription of inflammatory genes [19]. The inhibition of JAK tyrosine-kinases can block the intracellular signaling of a large number of cytokines involved in the pathophysiology of RA, such as IL-6 and IFN-γ [30]. In this context, it is also known that some of these cytokines, such as IL-6, are produced by muscle fibers (myokines). IL-6 could signal both atrophy/muscle degradation and hypertrophy/muscle regeneration, depending on the local conditions, such as acute or chronic exposure to the muscle fiber and the concomitant presence of other pro-inflammatory cytokines. Although during acute exercise IL-6 and consequently STAT signaling could lead to muscle regeneration, in an inflammatory environment, STAT downstream signals to a catabolic pathway [15]. Still, it has been shown that the blockade of JAK/STATs promotes satellite cell expansion [31] and the prevention of muscle wasting [16]. In our investigation, we observed increased levels of serum IL-6 in tofacitinib-treated mice. We speculate that this result shows a moment of a switch to the regeneration status signaling, in order to enhance muscle homeostasis. So, we investigated the effect of tofacitinib in the expression of myogenic markers such as MyoD, Myogenin, and Pax7, as well as the catabolism marker MurF1.

**Fig. 5:**
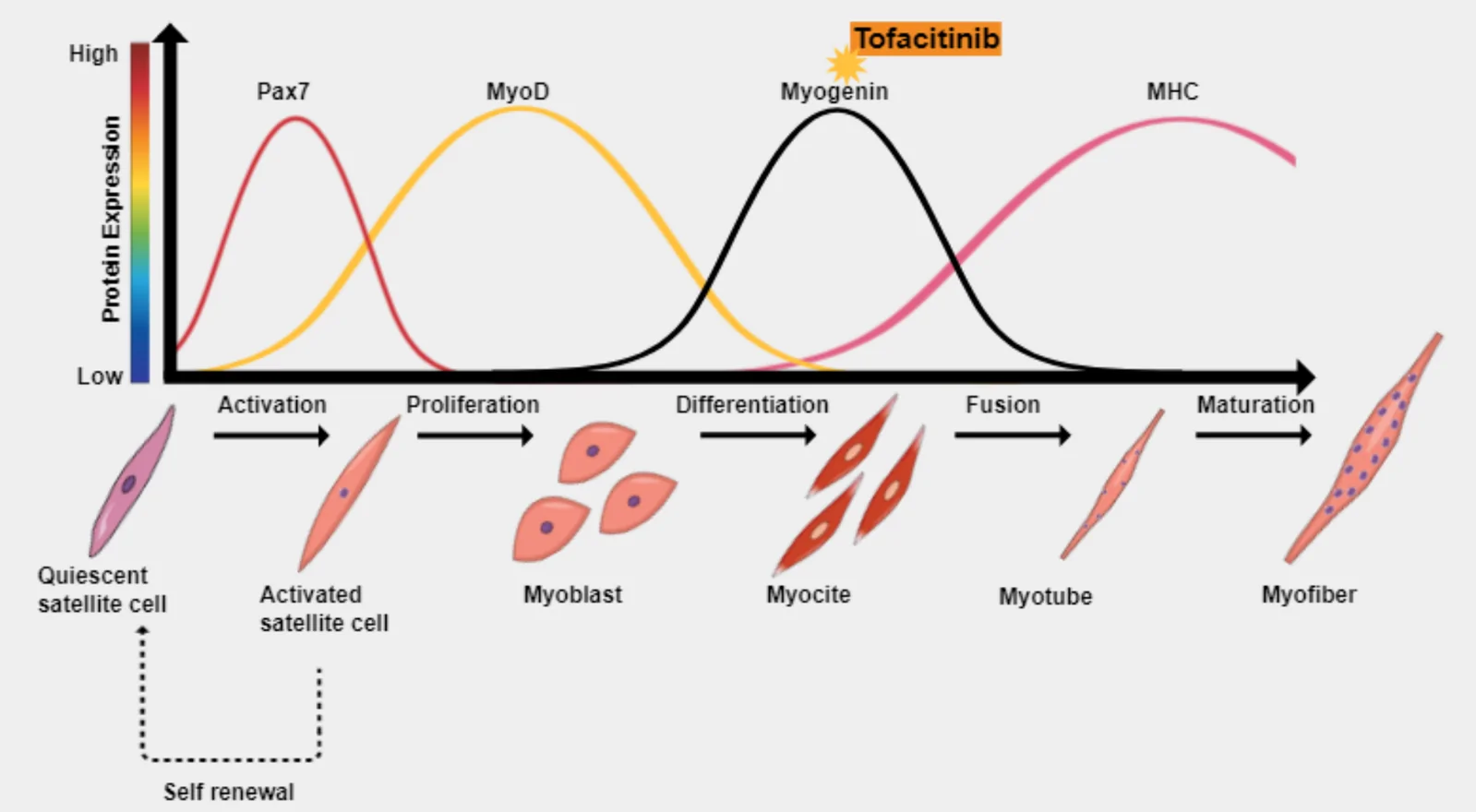
The possible effect of tofacitinib on muscle cell differentiation

Tofacitinib treatment did not change the pool of quiescent satellite cells in the GA muscle of arthritic mice as Pax7 expression remained similar to control and vehicle-treated groups. Although the UPS degradation of muscle mass was unchanged among groups, the muscle regeneration process showed increased differentiation of satellite cells, as myogenin had higher expression in tofacitinib-treated mice in comparison with control and vehicle-treated groups. In muscle biopsies of dermatomyositis patients treated with tofacitinib, a down-regulation of inflammatory genes, such as C1QB and C1QC, CXCL9, as well as type I IFN related genes, was detected [32]. This finding demonstrates that JAKs inhibition exerts an effect also locally in the muscle tissue. A previous study showed that the inhibition of the STAT pathway, specifically STAT3, was able to promote satellite cell expansion and acceleration of tissue repair [33]. Similarly, in elderly and dystrophic mice, the inhibition of either JAK or STAT3 expanded satellite cells and rescued muscle regeneration defects [34]. In our study, the evaluation of serum cytokines showed reduced TNF levels in tofacitinib-treated mice, which may contribute as a muscle regeneration stimulus, since the inhibition of TNF signaling also promotes myogenic commitment and satellite cell expansion [35, 36]. In an illustrative image (Fig. 5), we explain the possible effect of tofacitinib on muscle cell differentiation

To verify the effect of tofacitinib in sarcopenia, we measured both muscle mass and muscle function in mice. As we have previously described, the CIA model cause a state of impairment of both muscle mass and muscle function [22], findings that were reproduced in this study, as the vehicle group showed decreased body weight, muscle weight, muscle CSA, handgrip strength and fatigue (Fig. 2). Our study demonstrated, after treatment, an increase of 41.6%, and 69.4% in TA and GA weights, respectively. In RA patients, few studies assessed the effect of tofacitinib treatment on body composition and physical performance. Regarding body weight, an observational study addressed the effects of tofacitinib on body mass index (BMI). RA patients treated with tofacitinib for a year, had a median weight gain of 3 kg (4.2%), with 26% of the patients gaining 10% or more weight [37]. Therefore, there is little evidence of a significant effect of tofacitinib treatment on body composition of RA patients. In accordance with increased muscle regeneration after tofacitinib treatment, the muscle CSA of treated arthritic mice was also greater than the vehicle-treated group with an increase of 11.6%. Here, along with our muscle regeneration results, we speculate that the activation of myogenesis accounts for the increase in muscle mass in treated mice. Following the treatment, muscle strength was increased by 107.2% compared to the vehicle-treated group, showing handgrip strength similar to control mice. This result highlights the capability of tofacitinib in enhancing not only muscle weight but also muscle function. In the only record in the literature, a study showed improvement in muscle strength parameters in patients with juvenile dermatomyositis after 3 and 6 months of treatment with tofacitinib [38]. Although being a pilot, this study corroborates with our results, once our treatment reflects a long-term use of tofacitinib. However, more evidence is needed so that we can directly relate the treatment to increased strength in animals and patients. Regarding animal fatigue, we showed no differences between the treated groups. As the treated mice did not present a reduction in the nociception threshold, we believe it influenced their physical performance.

Briefly, we demonstrated that tofacitinib treatment was able to attenuate muscle loss in arthritic mice by modulating muscle regeneration. To the best of our knowledge, this is the first study about tofacitinib modulating muscle mass and physical performance. Considering our findings, especially the increased expression of myogenin, which is involved in the muscle anabolism pathway, we believe that tofacitinib could possibly improve muscle mass and strength muscle in RA patients. It is known that sarcopenia may directly impact physical function, life quality, and increased morbimortality. Therefore, randomized clinical trials with analysis of body composition and molecular analysis of muscle tissues could be performed to investigate the effects of the tofacitinib treatment on sarcopenia parameters.

However, our study showed some limitations. The evaluation of STATs protein expression, as well as pro-inflammatory cytokines, directly in the muscle could enrich the understanding of tofacitinib effects in this tissue. Also, to have a broader view of changes in body composition after treatment, an analysis of adipose tissue could be carried out. Further, the culture of muscle cells exposed to an inflammatory environment and the inhibition of JAKs is also an interesting approach to investigating the signaling pathways affected by the treatment. However, our findings show scientific relevance, once we detected positive effects of JAK inhibition in the muscle of arthritic mice, encouraging the performing of additional studies involving muscle molecular analysis in RA patients. Another limitation is that, with our results, we can not infer whether the modulation is direct -local action in the muscle - or indirect– by controlling inflammation Tofa improves myogenin expression and muscle regeneration.

In conclusion, tofacitinib treatment decreased muscle loss in arthritic mice. Tofacitinib was able to increase muscle weight and CSA in arthritic animals, together with increased expression of myogenin as a potential mechanism involved in that muscle gain. These results indicate tofacitinib as a possible candidate to treat RA consequent muscle waste.

## Electronic supplementary material

Below is the link to the electronic supplementary material.

Supplementary Material 1

## Data Availability

The authors confirm that the data supporting the findings of this study are available within the article and its supplementary materials.
