# Supplementary material for: Treatment with tofacitinib attenuates muscle loss through myogenin activation in the collagen-induced arthritis
Source: Adv Rheumatol. 2024 Nov 13;64:85. doi: 10.1186/s42358-024-00416-5 (PMC13488745; doi:10.1186/s42358-024-00416-5)
Supplement: Supplementary file 1 [file 42358_2024_416_MOESM1_ESM.docx]

**Supplementary Material**

**Treatment with tofacitinib attenuates muscle loss through myogenin activation in the collagen-induced arthritis.**

Thales Hein da Rosa, Bárbara Jonson Bartikoski, Rafaela Cavalheiro do Espírito Santo, Mirian Farinon, Jordana Miranda de Souza Silva, Renata Ternus Pedó, Maria Luísa Gasparini, Thais Karnopp, Leonardo Peterson dos Santos, Gustavo Chapacais, Andressa Di Domenico, Sofia Loch, Ricardo Machado Xavier.

**Supplementary Figure 1.** Histopathological score from experimental groups divided by 5 parameters of the joint: (A) Synovial inflammation, (B) Pannus, (C) Synovial hyperplasia, (D) Cartilage Erosion, (E) Bone erosion. Statistical analysis between groups was performed using ANOVA one-way followed by Bonferroni’s test. Data are presented as mean ± standard error of the mean (SEM). (F) Histopathology of joint confirmed articular damage of CIA animals by cartilage and bone erosion, synovial hyperplasia, invasive *pannus* formation, and inflammatory cell infiltrates.

**Supplementary Figure 2.** Normalization of (A) muscle strength, (B) TA and (C) GA weights by total body weight reveal that tofacitinib treatment prevent muscle loss. (D) Representative muscle histology stained with hematoxylin & eosin under 400x magnification.

**Supplementary Figure 3.** Original western blotting run of (A) PAX-7, (B) MyoD, (C) Miogenin, (D) MurF1 with the respective GAPDH run.

GAPDH

PAX7

A

GAPDH

MyoD

B

C

Miogenin

GAPDH

GAPDH

MurF-1

D
